# Diagnostic potential of myocardial early systolic lengthening for patients with suspected non-ST-segment elevation acute coronary syndrome

**DOI:** 10.1186/s12872-023-03364-y

**Published:** 2023-07-19

**Authors:** Wanwei Zhang, Qizhe Cai, Mingming Lin, Runyu Tian, Shan Jin, Yunyun Qin, Xiuzhang Lu

**Affiliations:** Department of Ultrasound Medicine, Beijing Chao Yang Hospital, Capital Medical University, Beijing, 100020 China

**Keywords:** Non-ST-segment elevation acute coronary syndrome, Early systolic lengthening, Speckle tracking echocardiography, Deformation

## Abstract

**Background:**

During early systole, ischemic myocardium with reduced active force experiences early systolic lengthening (ESL). This study aimed to explore the diagnostic potential of myocardial ESL in suspected non-ST-segment elevation acute coronary syndrome (NSTE-ACS) patients with normal wall motion and left ventricular ejection fraction (LVEF).

**Methods:**

Overall, 195 suspected NSTE-ACS patients with normal wall motion and LVEF, who underwent speckle tracking echocardiography (STE) before coronary angiography, were included in this study. Patients were stratified into the coronary artery disease (CAD) group when there was ≥ 50% stenosis in at least one major coronary artery. The CAD patients were further stratified into the significant (≥ 70% reduction of vessel diameter) stenosis group or the nonsignificant stenosis group. Myocardial strain parameters, including global longitudinal strain (GLS), duration of early systolic lengthening (DESL), early systolic index (ESI), and post-systolic index (PSI), were analyzed using STE and compared between groups. Receiver operating characteristic curve (ROC) analysis was performed to determine the diagnostic accuracy. Logistic regression analysis was conducted to establish the independent and incremental determinants for the presence of significant coronary stenosis.

**Results:**

The DESL and ESI values were higher in patients with CAD than those without CAD. In addition, CAD patients with significant coronary stenosis had higher DESL and ESI than those without significant coronary stenosis. The ROC analysis revealed that ESI was superior to PSI for identifying patients with CAD, and further superior to GLS and PSI for predicting significant coronary stenosis. Moreover, ESI could independently and incrementally predict significant coronary stenosis in patients with CAD.

**Conclusions:**

The myocardial ESI is of great value for the diagnosis and risk stratification of clinically suspected NSTE-ACS patients with normal LVEF and wall motion.

**Supplementary Information:**

The online version contains supplementary material available at 10.1186/s12872-023-03364-y.

## Introduction

Non-ST-segment elevation acute coronary syndrome (NSTE-ACS) accounts for more than 70% of acute coronary syndromes, and its incidence and mortality have been increasing for the past few decades [1]. Patients with suspected NSTE-ACS represent a heterogeneous population with substantial variations in the severity of coronary stenosis [2]. According to previous statistics, invasive protocols such as coronary angiography and revascularization therapy might be unnecessary in approximately one-third of these patients [3, 4]. However, the noninvasive identification of coronary artery lesions remains a clinical challenge, especially in suspected NSTE-ACS patients with normal wall motion and left ventricular ejection fraction (LVEF). Accordingly, continuous efforts are being made to develop reliable tools for the early detection of NSTE-ACS and the risk stratification of affected patients. The latest diagnostic and management guidelines for NSTE-ACS recommended the use of myocardial strain imaging to support the diagnosis of patients with clinically suspected ischemic disease but without wall motion abnormality [1]. Speckle tracking echocardiography (STE) can quantify the changes in myocardial deformation and accurately detect left ventricular (LV) dysfunction [5, 6]. Consequently, new and subtle deformational patterns have been introduced in this regard, such as myocardial early systolic lengthening (ESL), which is a promising marker of myocardial ischemia [7, 8].

**Fig. 1:**
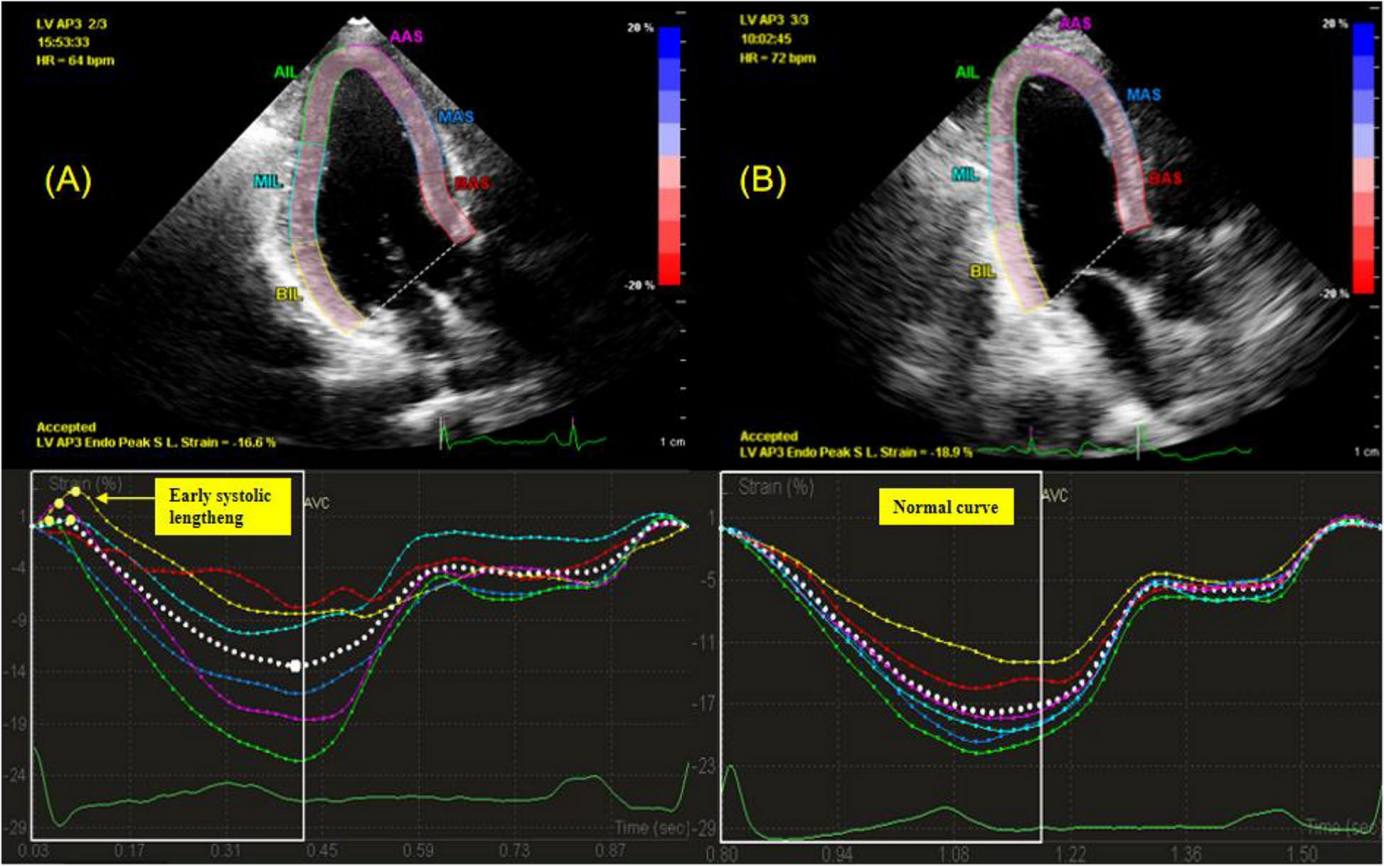
Left ventricular longitudinal strain profiles by speckle tracking echocardiography (STE). Representative image of a non-ST-segment elevation acute coronary syndrome (NSTE-ACS) patient with significant coronary stenosis and myocardial early systolic lengthening (ESL) (**A**); Representative image of a subject without coronary artery disease (CAD) showing no ESL (**B**)

Ischemic myocardial fibers with a reduced active force may exhibit stretching instead of shortening when the LV pressure rises during early systole. This paradoxical myocardial stretch is defined as ESL [9] (Fig. 1). Previous studies have revealed that ESL indicates subclinical contractile dysfunction and can be used to evaluate myocardial viability in patients with a higher risk of cardiovascular events [10–12]. However, the implications of ESL in patients with suspected NSTE-ACS have been only sparsely investigated. Hence, this study aimed to explore the diagnostic potential of myocardial ESL in suspected NSTE-ACS patients with normal LVEF and wall motion, and to determine its predictive value in the presence of significant coronary stenosis.

## Methods

### Study cohort

In this single-center study, 272 clinically suspected NSTE-ACS patients referred to coronary angiography from February to July 2019 were retrospectively enrolled. STE was performed within one day of coronary angiography. The following inclusion criteria were applied: LVEF ≥ 55% and no regional wall motion abnormality on conventional echocardiography. The exclusion criteria were as follows: (i) previous myocardial infarction, and/or revascularization procedure or cardiac resynchronization therapy; (ii) moderate or severe valve disease; (iii) cardiomyopathy and congenital heart disease; (iv) left bundle branch block or atrial fibrillation; (v) suboptimal echo images or ≥ 1 unanalyzable segment in the STE analysis. A total of 195 patients were finally included in this study (Supplemental Fig. 1). Patients with either unstable angina or non-ST elevation myocardial infarction (NSTEMI) exhibited symptoms and signs of myocardial ischemia at rest or with minimal exertion, whereas only those with the latter experienced acute cardiomyocyte injury/necrosis [1]. The baseline clinical characteristics of each patient on admission were retrieved from the hospital records. Body mass index (BMI) was calculated by dividing the body weight by the square meter of the patient height (kg/m^2^). Written informed consent was obtained from each patient, and the institutional ethics committee approved the research protocol (No. 2021-department-395).

### Echocardiography

Echocardiography was performed using the EPIQ system (version 7C, Philips Medical Systems, Bothell, Washington, USA) with an S5-1 phased-array transducer. Briefly, three consecutive heart cycles were obtained by two-dimensional images, and the recordings were digitally stored and subsequently analyzed offline using the Philips Qlab software (ver. 13.0). All patients underwent echocardiography before the coronary angiography (within three days of the initial admittance for NSTE-ACS). The echocardiographic data were analyzed by researchers blinded to all clinical information.

### Conventional echocardiographic analysis

The conventional echocardiography was evaluated according to the recommendations provided by the American Society of Echocardiography guidelines at the resting state [13]. The modified biplane Simpson’s method was used to calculate the LV end-systolic volume (LVESV) and the end-diastolic volume (LVEDV) at the apical 4- and 2-chamber views, and LVEF was subsequently calculated. LV volumes were indexed to body surface area to derive indexed LVEDV (LVEDVi) and indexed LVESV (LVESVi). The mitral inflow velocities of the peak early (E) and late (A) waves were obtained using pulsed-wave Doppler in the apical 4-chamber. Similarly, the peak early diastolic tissue velocity (e') was measured by tissue Doppler imaging in the septal and lateral mitral annulus. Then, the e' value was averaged, and the E/e’-ratio was calculated.

### STE and ESL analysis

**Fig. 2:**
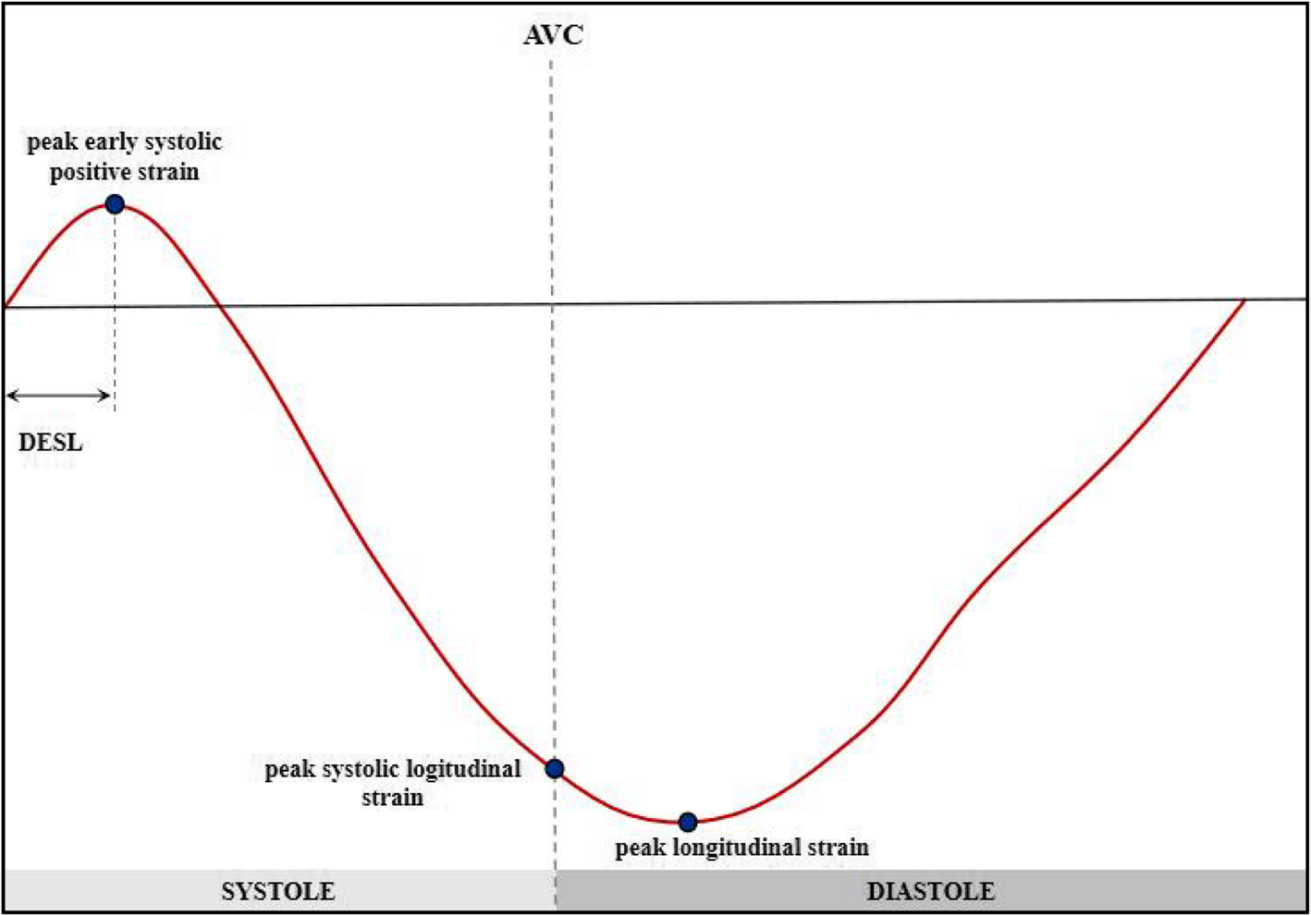
Schematic drawing of early systolic lengthening (ESL) (*red curve*). The duration of early systolic lengthening (DESL) was defined as the time from end-diastole to peak early systolic positive strain. The early systolic index (ESI) was calculated as follows: ESI = -100 × (peak early systolic positive strain/peak longitudinal strain). The post-systolic index (PSI) was calculated as [100 × (peak longitudinal strain–peak systolic longitudinal strain)/peak longitudinal strain]. AVC, aortic valve closure

Two-dimensional echocardiographic images of the apical 2-, 3-, and 4-chambers were used to determine the LV longitudinal strain. The frame rate was set at 65 ± 7 frames/s. In each apical view, a region of interest was automatically traced by the software, with subsequent manual adjustment to ensure accurate tracking, if necessary. End-systole was defined as the time of aortic valve closure (AVC). From the STE curves, the following strain values were extracted in each segment: peak early systolic positive strain (indicating maximum myocardial lengthening during early systole), peak systolic longitudinal strain (indicating maximum systolic shortening), and peak longitudinal strain (indicating maximum shortening in cardiac cycle). As a measure of global systolic function, the global longitudinal strain (GLS) was calculated by averaging the peak systolic strain values from an 18-segment model. The standard deviation of the time to peak systolic strain in 18 segments of the left ventricle represents the mechanical dispersion (MD). We defined the duration of ESL (DESL) as the time from end-diastole (peak R-wave of the electrocardiogram) to early systolic strain. We assessed the early systolic index (ESI) as [-100 × (peak early systolic positive strain/peak longitudinal strain)] (Fig. 2), which was previously investigated as the “lengthening/shortening ratio” [9]. Post-systolic shortening (PSS) was defined as persistent shortening beyond AVC. The post-systolic index (PSI) was calculated as [100 × (peak longitudinal strain–peak systolic longitudinal strain)/peak longitudinal strain]. Zero was assigned to the corresponding parameters when ESL or PSS was not present. The values were averaged across 18 segments to obtain the global value per patient. Based on the 18-LV segment model of myocardial perfusion territories [14], the territorial strain was calculated as the average value of the segments belonging to the perfusion areas of the left anterior descending artery (LAD), left circumflex artery (LCX), or right coronary artery (RCA), respectively.

### Coronary angiography

Coronary angiography was performed using a standard technique, which achieved digital image acquisition and storage. The patients were stratified into the coronary artery disease (CAD) group when there was ≥ 50% stenosis in at least one major coronary artery. No CAD was defined as the group with any main coronary stenosis < 50% or normal coronary arteries. Patients with CAD were further stratified into the significant (≥ 70% reduction of vessel diameter) stenosis group or the nonsignificant stenosis group. CAD patients were also grouped based on the number of significantly stenosed arteries. For patients with significant coronary stenosis, percutaneous coronary intervention (PCI) was performed based on the judgment of the operator.

### Statistical analysis

Continuous variables were expressed as the mean ± standard deviation or median (25^th^–75^th^ percentiles), depending on whether the data were normally distributed. Between-group comparisons were analyzed by the Student’s *t*–test, Mann–Whitney U test, analysis of variance, or Kruskal–Wallis test, with Bonferroni correction applied for post hoc tests as appropriate. Categorical variables were presented as numbers (percentages), and the chi-squared or Fisher’s exact test was performed to compare the differences between groups. Diagnostic accuracy was assessed by calculating the area under the receiver operating characteristic (ROC) curve (AUC). The comparison between AUCs was tested using the method of DeLong in MedCalc. Spearman’s rho was used to evaluate the correlation between the LV deformational parameters. Logistic regression analysis was conducted to determine the association between the baseline clinical characteristics, echocardiographic variables, and significant coronary stenosis in NSTE-ACS patients. In addition, the potential incremental values of the strain parameters in terms of diagnostic utility were assessed by comparing the AUCs of a series of prediction models. Intra-observer and inter-observer variabilities were assessed for the LV deformation parameters using intraclass correlation coefficients (ICCs). The analyses were performed by two independent observers in 10 randomly selected patients. A *P*-value of < 0.05 was considered statistically significant. The statistical analyses were performed using SPSS version 23.0 (SPSS, Chicago, IL, USA) and MedCalc 15.6 (MedCalc Software, Ostend, Belgium).

## Results

### Baseline characteristics

A total of 195 patients with suspected NSTE-ACS (mean age: 63 ± 9 years, male: 56%) were included in the present study. Based on the coronary angiographic findings, all patients were initially divided into two groups: no CAD (*n* = 30) and CAD (*n* = 165). The patients with CAD were then subdivided into the significant stenosis group (*n* = 122) or the nonsignificant stenosis group (*n* = 43). The detailed baseline characteristics are summarized in Table 1. Among the patients with CAD, 154 patients (93%) had unstable angina and 11 patients (7%) had NSTEMI. The patients with CAD were predominantly male, had higher rates of diabetes mellitus, and had higher levels of troponin I compared to patients without CAD (*P* < 0.05). In the subgroups of CAD, the patients with significant coronary stenosis had a higher prevalence of diabetes mellitus and higher levels of troponin I compared with those in the nonsignificant stenosis group (*P* < 0.05). There were no signifcant differences in the other clinical characteristics among the no CAD group, nonsignificant stenosis group and significant stenosis group.

**Table 1:** Baseline characteristics of suspected NSTE-ACS patients

Variable	No CAD (*n* = 30)	CAD (*n* = 165)	Nonsignificant stenosis (*n* = 43)	Significant stenosis (*n* = 122)
Clinical variables				
Age, years	61 ± 7	64 ± 9	64 ± 9	64 ± 9
Male	9 (30%)	100 (61%)^*^	26 (60%)^*^	74 (61%)^*^
BMI	25.2 ± 3.3	25.4 ± 3.3	25.6 ± 3.2	25.3 ± 3.4
SBP, mmHg	131 ± 14	135 ± 17	138 ± 20	135 ± 16
DBP, mmHg	73 ± 9	76 ± 11	78 ± 12	76 ± 10
Heart rate, beats/min	72 ± 15	73 ± 11	72 ± 11	73 ± 10
Hypertension	16 (53%)	119 (72%)	28 (65%)	91 (75%)
Dyslipidemia	25 (83%)	146 (89%)	39 (91%)	107 (88%)
Diabetes mellitus	7 (23%)	67 (41%)^*^	11 (26%)	56 (46%)^*△^
Smoking	11 (37%)	69 (42%)	17 (40%)	52 (43%)
Family history of CAD	7 (23%)	33 (20%)	12 (28%)	21 (17%)
Troponin I, ng/L	0.0 (0.0, 0.0)	0.0 (0.0, 50.0)^*^	0.0 (0.0, 0.0)	10 (0.0, 295.0)^*△^
Diseased vessel				
LAD	0 (0%)	152 (92%)	36 (84%)	116 (95%)
LCX	0 (0%)	92 (56%)	10 (23%)	82 (67%)
RCA	0 (0%)	114 (69%)	24 (56%)	90 (74%)
Revascularization	0 (0%)	110 (67%)	0 (0%)	110 (90%)

*NSTE-ACS* Non-ST-segment elevation acute coronary syndrome, *CAD* Coronary artery disease, *BMI* Body mass index, *SBP* Systolic blood pressure, *DBP* Diastolic blood pressure, *LAD* Left anterior descending artery, *LCX* Left circumflex coronary artery, *RCA* Right coronary artery ^*^P < 0.05 vs. no CAD group; ^△^*P* < 0.05 vs*.* significant stenosis group

### Conventional echocardiography and LV myocardial deformation

The comparisons of the conventional echocardiographic findings and the global LV myocardial deformation parameters are presented in Table 2. No significant differences in LV volumes, LVEF or MD were observed among the three groups. The patients with CAD had a mean LVEF of 62%, suggesting normal LV systolic function, but had a higher E/e' ratio than those with no CAD (*P* < 0.05). Compared to the patients without CAD, those with CAD had lower absolute GLS (*P* < 0.05) values and higher DESL, ESI, and PSI values (*P* < 0.05). Notably, the patients in the significant stenosis group had markedly higher DESL, ESI, and PSI values as well as impaired GLS compared to the subjects in the other two groups (*P* < 0.05). The values of all strain parameters followed the trend of the no CAD group, the nonsignificant stenosis group, and the significant stenosis group. Comparisons among patients with CAD according to the number of significantly stenosed coronary arteries are shown in Supplemental Table 1. The patients with one or more vessels with significant coronary stenosis had higher DESL and ESI values than those without significant coronary artery stenosis. Poor correlations were found between GLS and the ESL parameters (Spearman’s rho ≤ 0.20). PSI correlated moderately with DESL (Spearman’s rho = 0.39), which also was the case for ESI (Spearman’s rho = 0.49) (Supplemental Table 2). Regarding the regional strain, there were statistically significant differences of DESL and ESI between segments of the arteries with and without significant stenosis for each considered coronary territory (*P* < 0.05, Supplemental Table 3).

**Table 2:** Echocardiographic findings for suspected NSTE-ACS patients

Variable	No CAD (*n* = 30)	CAD (*n* = 165)	Nonsignificant stenosis (*n* = 43)	Significant stenosis (*n* = 122)
LVEDV, mL	75.3 ± 16.6	74.2 ± 17.3	74.8 ± 15.5	73.7 ± 18.1
LVESV, mL	27.3 ± 6.4	28.4 ± 7.7	27.9 ± 6.4	28.8 ± 8.4
LVEDVi, mL/m2	43.9 ± 10.6	42.2 ± 9.1	42.5 ± 8.5	42.1 ± 9.3
LVEDVi, mL/m2	16.0 ± 4.2	16.3 ± 4.3	15.8 ± 3.1	16.4 ± 4.6
LVEF, %	62.7 ± 1.8	61.5 ± 3.6	62.3 ± 3.6	61.3 ± 3.5
E/e’ ratio	9.7 ± 2.7	11.40 ± 3.7^*^	11.5 ± 3.8	11.7 ± 3.8^*^
GLS, %	-19.30 ± 1.60	-17.80 ± 2.40^*^	-18.50 ± 2.20	-17.60 ± 2.40^*△^
MD, ms	16.2 (6.6, 25.1)	15.7 (8.0, 22.3)	15.7 (7.4, 22.9)	16.0 (8.0, 31.3)
PSI, %	1.00 (0.44, 3.64)	2.09 (1.01, 4.84)^*^	1.52 (0.61, 2.59)	2.69 (1.28, 6.49)^*△^
DESL, ms	4.5 (1.6, 8.1)	9.7 (5.6, 14.4)^*^	6.3 (1.9, 8.9)	11.5 (7.5, 15.6)^*△^
ESI, %	0.32 (0.07, 0.54)	0.91 (0.44, 2.06)^*^	0.42 (0.07, 0.79)	1.34 (0.67, 2.61)^*△^

*NSTE-ACS* Non-ST-segment elevation acute coronary syndrome, *CAD* Coronary artery disease, *LVEDV* Left ventricular end-diastolic volume, *LVESV* Left ventricular end-systolic volume, *LVEDVi* Indexed left ventricular end-diastolic volume, *LVESVi* Indexed left ventricular end-systolic volume, *LVEF* Left ventricular ejection fraction, *GLS* Global longitudinal strain, *MD* Mechanical dispersion, *PSI* Post-systolic index, *DESL* Duration of early systolic lengthening, *ESI* Early systolic index

^*^P < 0.05 vs. no CAD group; ^△^*P* < 0.05 vs. significant stenosis group

**Table 3:** Results of ROC to detect patients with CAD and significant coronary stenosis

**Variable**	Patients with CAD				Significant coronary stenosis			
	AUC	Cut-off	sensitivity (%)	specificity (%)	AUC	Cut-off	sensitivity (%)	specificity (%)
GLS, %	0.69	-17.3	38	97	0.61	-18.9	73	49
PSI, %	0.65	1.0	75	57	0.69	3.20	46	91
DESL, ms	0.75	8.7	56	87	0.78^*^	9.0	66	77
ESI, %	0.78^△^	0.6	65	80	0.81^*△^	1.0	62	88

*ROC* Receiver operating characteristic curve, *CAD* Coronary artery disease, *AUC* Area under the curve, *GLS* Global longitudinal strain, *PSI* Post-systolic index

^*^P < 0.05 vs. GLS; ^△^*P* < 0.05 vs*.* PSI

### Diagnostic ability of myocardial ESL to identify CAD and significant coronary stenosis

**Fig. 3:**
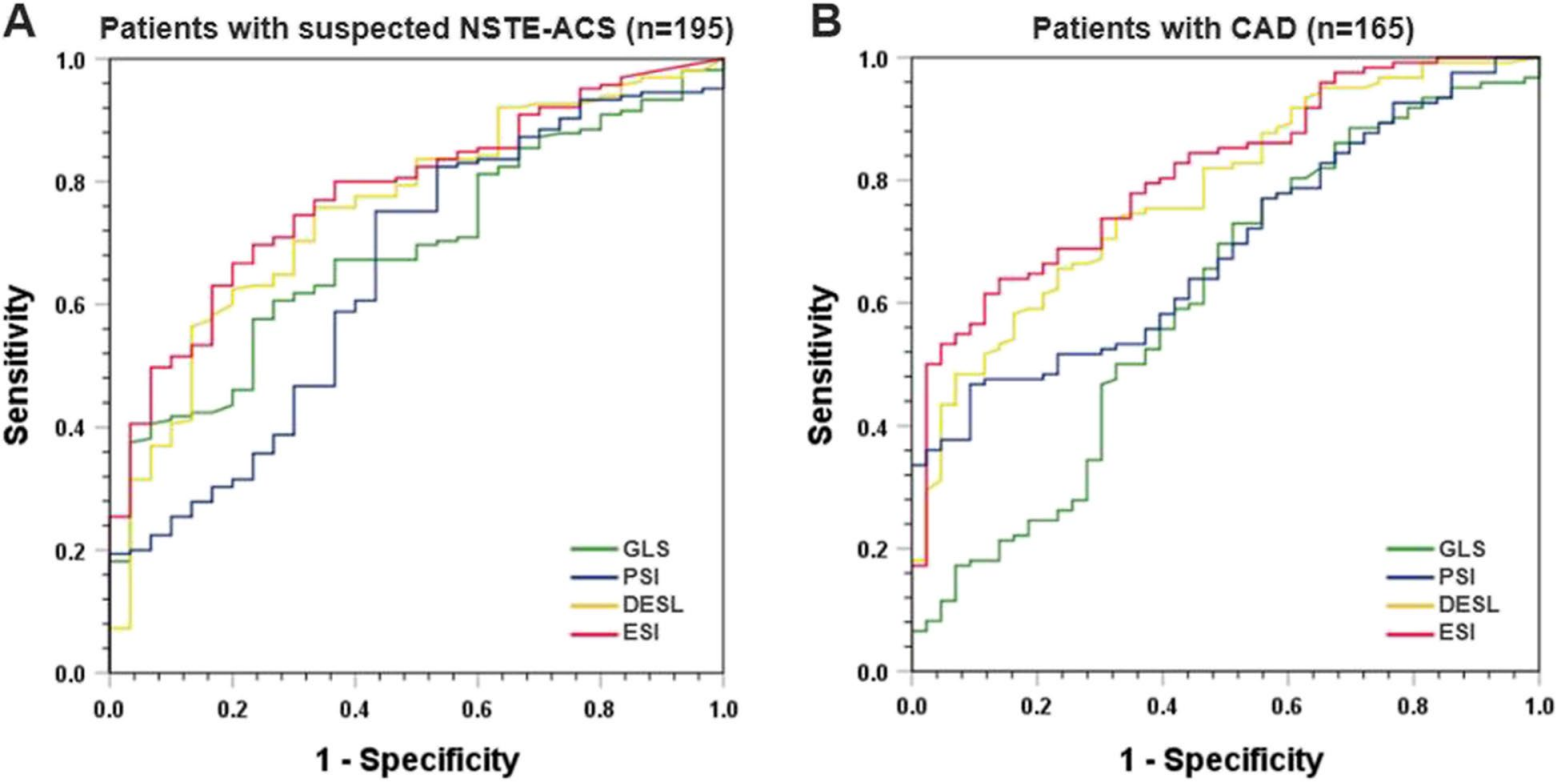
The ROC curves for detecting (**A**) patients with CAD and (**B**) significant coronary stenosis. The early systolic index (ESI) was superior to the post-systolic index (PSI) for identifying patients with CAD among suspected non-ST-segment elevation acute coronary syndrome (NSTE-ACS) patients. The ESI was superior to the global longitudinal strain (GLS) and the PSI for discriminating between CAD patients with and without significant coronary stenosis

The myocardial ESI had a superior ability than PSI to identify patients with CAD among suspected NSTE-ACS patients (AUC: 0.78 vs. 0.69, *P* < 0.05); Table 3, Fig. 3A), with a sensitivity of 65% and a specificity of 80%. Furthermore, ESI was superior to GLS (AUC: 0.81 vs. 0.61, *P* < 0.05) and PSI (AUC: 0.81 vs. 0.69, *P* < 0.05) in discriminating between significant and nonsignificant coronary stenosis in patients with CAD, providing a sensitivity of 62% and a specificity of 88% (Table 3, Fig. 3B). In addition, the AUCs for identifying significant stenosis of the LAD, LCX, and RCA were the greatest for the territorial ESI, although the diagnostic accuracy was moderate (AUCs ranging between 0.60 and 0.65, Supplemental Table 3).

### Logistic regression models for significant coronary stenosis prediction

**Fig. 4:**
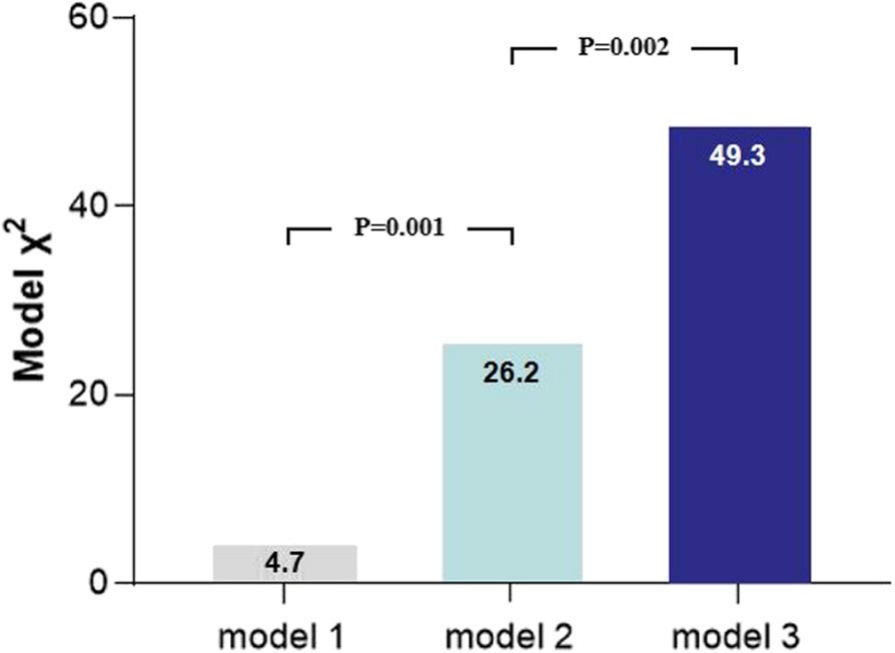
Incremental value of the early systolic index (ESI) in a series of logistic regression models for the prediction of significant coronary stenosis in patients with CAD. (Model 1: global longitudinal strain (GLS); model 2: model 1 plus the post-systolic index (PSI); model 3: model 2 plus the ESI)

Table 4 presents the logistic regression analysis results for identifying the independent predictors of significant coronary stenosis in patients with CAD. DESL was excluded from the multivariate analysis due to its close relationship with ESI. After the multivariate adjustment, ESI remained the only independent predictor of significant coronary stenosis (odds ratio [OR]: 3.31, 95% confidence interval [CI]: 1.55–7.04; *P* = 0.002). As shown in Fig. 4, the inclusion of PSI provided an incremental benefit over GLS (model 1) for predicting significant coronary stenosis in patients with CAD (*χ*^*2*^ = 26.2 vs. 4.7, *P* = 0.001). Furthermore, the predictive efficacy of model 2 (model 1 plus PSI) was significantly improved by adding ESI (model 3), with an increase in *χ*^*2*^ value from 26.2 to 49.3 (*P* = 0.002).

**Table 4:** Logistic regression analyses to predict significant coronary stenosis

Variable	Univariate logistic regression			Multivariate logistic regression		
	**OR**	**95% CI**	***P***	**OR**	**95% CI**	***P***
DM	0.41	0.19–0.88	0.022	0.57	0.22–1.46	0.241
Troponin I	1.06	1.00–1.13	0.043	1.05	0.99–1.12	0.104
GLS	1.19	1.01–1.39	0.035	1.14	0.92–1.41	0.236
PSI	1.45	1.18–1.77	< 0.001	1.16	0.93–1.44	0.180
ESI	5.10	2.44–10.65	< 0.001	3.31	1.55–7.04	0.002

*OR* Odds ratio, *CI* Confidence interval, *DM* Diabetes mellitus, *GLS* Global longitudinal strain, *PSI* Post-systolic index, *ESI* Early systolic index

### Reproducibility

The ICCs of inter- and intra-observer variability were 0.83 and 0.95 for GLS, and 0.62 and 0.85 for PSI, respectively. Furthermore, moderate reproducibility for DESL (0.87 and 0.62 for intra- and inter-observer ICC, respectively) and ESI (0.85 and 0.59 for intra- and inter-observer ICC, respectively) were identified in the present study (Supplemental Table 4).

## Discussion

The main findings of the present study were as follows: (i) DESL and ESI were higher in the patients with CAD than in those without CAD; furthermore, the patients in the significant stenosis group had higher DESL and ESI values than those in the nonsignificant coronary group. (ii) ESI was superior to PSI for identifying patients with CAD, and it was further superior to GLS and PSI for predicting the presence of significant coronary stenosis. (iii) ESI could provide independent and incremental value for predicting significant coronary stenosis in patients with CAD. These results suggest that myocardial ESL may be valuable for the diagnosis and risk stratification of clinically suspected NSTE-ACS patients with normal LVEF and wall motion. 

A previous study has confirmed that up to one-third of suspected NSTE-ACS patients may not need coronary angiography [4]. In patients with significant coronary stenoses and unacceptable angina pectoris, the use of PCI to improve symptoms is reasonable [15]. Accordingly, the development of robust diagnostic tools to better select patients requiring invasive procedures could reduce the heavy burden on healthcare costs and complication rates. Given that LVEF and wall motion may be normal during chest pain-free periods [16], traditional echocardiography alone is not sufficient to diagnose patients with suspected NSTE-ACS. The latest guidelines for the diagnosis and management of NSTE-ACS suggest that myocardial strain imaging can support the diagnosis of patients with clinical suspicion of ischemic disease and without wall motion abnormality [1]. With the introduction of STE, new attention has been paid to paradoxical myocardial deformation, including ESL. This phenomenon represents a passive myocardial stretch in early systole, which is considered as a sensitive marker of myocardial ischemia [17, 18], and provides useful information for risk stratification in various diseases [19–21]. In particular, ESI has emerged as a novel tool to detect myocardial ischemia memory [22] and viable myocardium [17, 23]. However, there is limited data on the diagnostic potential of myocardial ESL in suspected NSTE-ACS patients.

The present study revealed that DESL and ESI were higher in patients with CAD compared to those without CAD. Furthermore, patients in the significant stenosis group had higher ESL values compared to those in the nonsignificant stenosis group. These findings suggest that myocardial ESL can be used to detect minor LV systolic dysfunction earlier than the LVEF and wall motion visual evaluation. Consistent with Weng et al. [6], there was no significant difference in MD between the patients with significant and nonsignificant coronary stenosis in this study. It was considered that the study population included suspected NSTE-ACS patients with normal LVEF, no wall motion abnormality, and no previous history of myocardial infarction, which might not have severe pathological changes associated with discrete mechanics. ESL may be associated with LV desynchrony [22], often occurring in the myocardium with systolic dysfunction, and increasing with the severity of myocardial ischemia [24, 25], which is consistent with the results of this study. Several mechanisms have been proposed to explain the paradoxical myocardial stretch observed in CAD. Moreover, myocardial recurrent ischemic insults caused by coronary flow disturbance may lead to structural changes, such as fibrosis, which may affect the segmental interactions and regional deformation [26]. In addition, there is a “tug-of-war” effect among LV segments during isovolumic contraction [27, 28]. Accordingly, a normal myocardium would create a sufficient active force to shorten, while an ischemic myocardium with a reduced active force would passively lengthen when the LV pressure increases during early systole. As expected, the regional ESL parameters significantly increased in segments correlated to significant coronary stenosis compared to the nonsignificant stenotic segments for each coronary territory.

Caspar et al. [29] found that GLS displayed good diagnostic performance to predict CAD (AUC = 0.92) in suspected NSTE-ACS patients with normal systolic function. In our study, GLS had a moderate ability to identify patients with CAD (AUC = 0.69), while it was inferior to ESI (AUC: 0.61 vs. 0.81, *P* < 0.05) for predicting significant coronary stenosis. The different capacity of GLS to diagnose CAD may be due to the less severe myocardial damage in the CAD patients with normal LVEF and wall motion in our study. Even in this situation, ESI still had excellent diagnostic performance, assumingly because the calculation of ESI integrated both peak early systolic positive strain and peak longitudinal strain in the cardiac cycle equally. Several reasons may contribute to this result. First, GLS reflects only the average level of the peak systolic longitudinal strain, while ESI integrates both the early systolic peak positive strain and the peak systolic longitudinal strain. In addition, GLS cannot fully reflect mechanical systolic motion, and the coordination of the LV systolic motion can be analyzed by ESL. Therefore, ESI was a more reliable marker of myocardial function in suspected NSTE-ACS patients with normal LVEF and wall motion.

Some researchers have suggested that PSS, a delayed myocardial contraction after end-systole, is equal or more important than the commonly used LV strain in patients with CAD [30, 31]. Consistent with the study of Weng et al. [6], we found that the addition of PSI to GLS was associated with improved prediction of significant coronary stenosis. PSS and ESL are known as paradoxical motions caused by the imbalance of tension between the ischemic and non-ischemic myocardium [7]. Notably, ESI exhibited a superior diagnostic efficacy for CAD and the presence of significant coronary stenosis compared to that of PSI. On the one hand, ischemia-related myocardial injuries lead to heterogeneity in tissue contraction and induce ESL, which might represent the first minor change in myocardial function, thus affecting the PSS via passive elastic recoil. On the other hand, ESL may exert a partial compensatory effect on systolic strain via the Frank-Starling mechanism, thus influencing PSS. Therefore, ESL may be more susceptible to pathologic changes and potential ischemia. Similar to Ishigaki et al. [32], we also found that ESI provided incremental value over GLS and PSI for predicting significant coronary stenosis in patients with CAD. Since myocardial ESL can easily be quantified using STE, this novel approach can exhibit a conveniently synergistic role in clinical practice. Our results supported the use of the ESI as an independent predictor because it may be a unique early marker of ischemia-induced pathological alterations and myocardial dysfunction. Taken together, ESI may be a more valuable tool for the identification and risk stratification of patients with NSTE-ACS compared to GLS and PSI.

However, the cut-off values for ESL-related indices should be interpreted with caution, since the values in our study were markedly lower than those from a previous study [24]. We considered a few potential explanations. In terms of methodology, both studies used 18-segment models of the left ventricle; therefore, this probably did not influence the results. However, the two study populations differed, with our study including patients with NSTE-ACS and the other studies including patients with stable CAD [12, 33]. In addition, we should consider the vendor-related variability in two-dimensional STE measures and the difference in the framerates between the present study and the previous study. The ability of regional ESL parameters to identify significant lesions of the respective coronary territory was moderate. This can be explained by the possibility of anatomical variations in the coronary arteries and perfusion in both arteries, making rigorous regional analysis somewhat inaccurate. Future large-scale prospective studies are warranted to validate these findings.

Our study had some limitations that must be addressed. First, this was a retrospective study with a relatively small number of subjects; thus, large-scale and prospective studies are needed to confirm these results. Second, the radial and circumferential strains were not analyzed because longitudinal mechanics were sensitive markers of cardiovascular disease [34]. However, there were no significant differences in the ESL analysis among all directions [25]. Third, several previous studies determined the starting point of ESL as the time from onset of the Q-wave on the electrocardiogram; however, the software (Qlab 13.0) allows it to be set only in one particular frame (peak R-wave of the electrocardiogram). Fourth, measurements of fractional flow reserve were not taken in all patients, so future studies should investigate the association between a functionally significant lesion and ESL. Finally, there was a lack of long-term patient information at the follow-up after PCI, and no special tracking analysis was performed. Therefore, further investigations are warranted to elucidate whether myocardial ESL can predict the functional improvement after PCI and determine its potential prognostic value in NSTE-ACS patients.

## Conclusions

The myocardial ESI could identify patients with CAD suspected of having NSTE-ACS and further accurately predict the presence of significant coronary stenosis. In addition, this index provided an independent and incremental predictive ability for significant coronary stenosis in patients with CAD. Therefore, the myocardial ESI may be a potentially valuable tool for the diagnosis and risk stratification of suspected NSTE-ACS patients without wall motion or LVEF abnormality.

## Supplementary Information

**Additional file 1: Supplemental Fig 1. **Flowchart of the study population.

**Additional file 2.** **Supplemental Table 1. **Echocardiographic data according to the extent of CAD.

**Additional file 3.** **Supplemental Table 2. **Spearman’s correlations between myocardial deformational parameters.

**Additional file 4.** **Supplemental Table 3. **ROC curve results to predict significant coronary branch stenosis.

**Additional file 5.** **Supplemental Table 4. **Interobserver and intraobserver variability (ICCs).

## Data Availability

The datasets analyzed in this study are available from the corresponding author on reasonable request.

## Abbreviations

BMI: Body Mass Index
CAD: Coronary Artery Disease
DESL: Duration of Early Systolic Lengthening
ESI: Early Systolic Index
ESL: Early Systolic Lengthening
GLS: Global Longitudinal Strain
ICC: Intraclass Correlation Coefficient
LAD: Left Anterior Descending Artery
LCX: Left Circumflex Artery
LV: Left Ventricular
LVEF: Left Ventricular Ejection Fraction
MD: Mechanical Dispersion
NSTE-ACS: Non-ST-segment Elevation Acute Coronary Syndrome
NSTEMI: Non-ST elevation Myocardial Infarction
PSI: Post-systolic Index
PSS: Post-systolic Shortening
RCA: Right Coronary Artery
STE: Speckle Tracking Echocardiography
