# Supplementary material for: Diagnostic potential of myocardial early systolic lengthening for patients with suspected non-ST-segment elevation acute coronary syndrome
Source: BMC Cardiovasc Disord. 2023 Jul 19;23:364. doi: 10.1186/s12872-023-03364-y (PMC10357602; doi:10.1186/s12872-023-03364-y)

Additional Material

Diagnostic potential of myocardial early systolic lengthening for patients with suspected non-ST-segment elevation acute coronary syndrome

Wanwei Zhang[1], Qizhe Cai[1], Mingming Lin[1], Runyu Tian[1], Shan Jin[1], Yunyun Qin[1,*], Xiuzhang Lu[1,*]

[1]Department of Ultrasound Medicine, Beijing Chao Yang Hospital, Capital Medical University, Beijing, 100020, China.

***Corresponding authors:**

Yunyun Qin, MD, PhD:
Department of Ultrasound Medicine, Beijing Chao Yang Hospital, Capital Medical University, Beijing, 100020, China.
Email: yun_23@126.com

Xiuzhang Lu, MD, PhD:
Department of Ultrasound Medicine, Beijing Chao Yang Hospital, Capital Medical University, Beijing, 100020, China.
Email: echolxz @163.com

Supplemental Fig.1 Flowchart of the study population

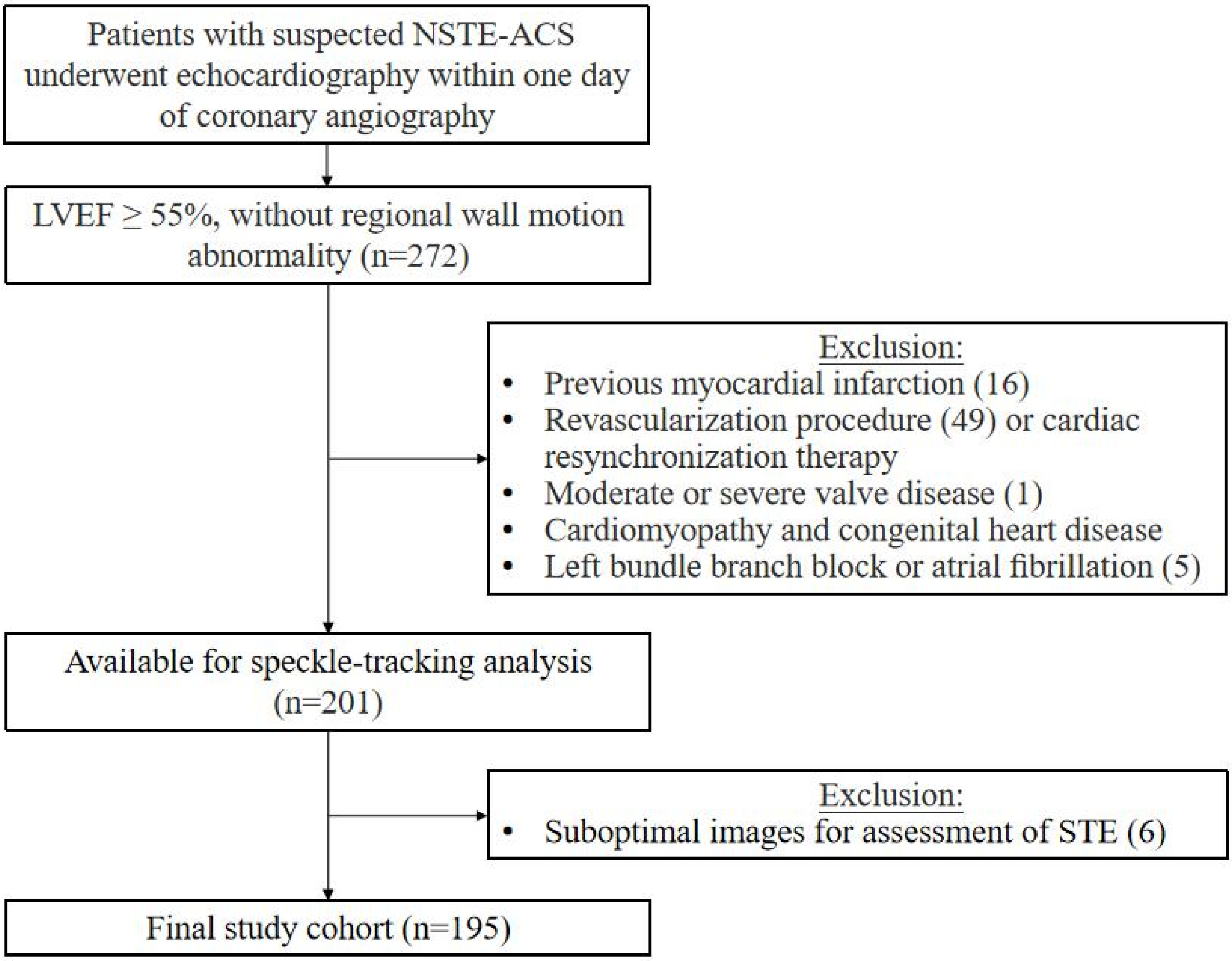

Supplement: Supplementary file 1 — Additional file 1: Supplemental Fig 1. Flowchart of the study population. [file 12872_2023_3364_MOESM1_ESM.pdf]
