# Supplementary material for: Diagnostic potential of myocardial early systolic lengthening for patients with suspected non-ST-segment elevation acute coronary syndrome
Source: BMC Cardiovasc Disord. 2023 Jul 19;23:364. doi: 10.1186/s12872-023-03364-y (PMC10357602; doi:10.1186/s12872-023-03364-y)
Supplement: Supplementary file 2 — Additional file 2. Supplemental Table 1. Echocardiographic data according to the extent of CAD. [file 12872_2023_3364_MOESM2_ESM.pdf]

Additional Material

Diagnostic potential of myocardial early systolic lengthening for patients with suspected non-ST-segment elevation acute coronary syndrome

Wanwei Zhang[1], Qizhe Cai[1], Mingming Lin[1], Runyu Tian[1], Shan Jin[1], Yunyun Qin[1,*], Xiuzhang Lu[1,*]

[1]Department of Ultrasound Medicine, Beijing Chao Yang Hospital, Capital Medical University, Beijing, 100020, China.

***Corresponding authors:**

Yunyun Qin, MD, PhD:

Department of Ultrasound Medicine, Beijing Chao Yang Hospital, Capital Medical University, Beijing, 100020, China.

Email: yun_23@126.com

Xiuzhang Lu, MD, PhD:

Department of Ultrasound Medicine, Beijing Chao Yang Hospital, Capital Medical University, Beijing, 100020, China.

Email: echolxz @163.com

Supplemental Table 1. Echocardiographic data according to the extent of CAD

Variable	No Significant stenosis (*n*=43)	Single vessel (*n*=60)	Two vessels (*n*=39)	Three vessels (*n*=23)
LVEDV, mL	74.8±15.5	74.5±19.8	71.0±17.3	76.4±14.7
LVESV, mL	27.9±6.4	29.1±9.5	27.9±7.3	29.9±7.4
LVEDVi, mL/m2	42.5±8.5	42.5±10.2	40.7±8.9	43.3±7.6
LVEDVi, mL/m2	15.8±3.1	16.5±5.2	16.0±4.1	16.3±4.3
LVEF, %	62.3±3.6	61.8±3.8*	60.8±3.2*	61.3±3.6*
E/e' ratio	11.5±3.8	11.2±3.9	11.8±4.1	12.9±3.2
GLS, %	-18.50±2.20	-17.83±2.56	-17.75±1.98	-16.86±2.46*
MD, ms	15.7 (7.4, 22.9)	15.8 (5.8, 21.0)	16.7 (11.0, 24.4)	17.6 (7.7, 23.1)
PSI, %	1.52 (0.61, 2.59)	2.03 (0.93, 5.01)	2.40 (1.14, 6.92)*	4.28 (1.95, 7.65)*
DESL, ms	6.3 (1.9, 8.9)	10.5 (7.3, 15.7)*	11.5 (6.6, 15.5)*	13.1 (9.7, 16.9)*
ESI, %	0.42 (0.07, 0.79)	1.08 (0.54, 2.51)*	1.59 (0.67, 3.33)*	1.49 (0.95, 2.39)*

CAD, coronary artery disease; LVEDV, left ventricular end-diastolic volume; LVESV, left ventricular end-systolic volume; LVEDVi, indexed left ventricular end-diastolic volume; LVESVi, indexed left ventricular end-systolic volume; LVEF, left ventricular ejection fraction; GLS, global longitudinal strain; MD, mechanical dispersion; PSI, post-systolic index; DESL, duration of early systolic lengthening; ESI, early systolic index. $^*P<0.05$ vs. no significant stenosis group.
