# Supplementary material for: Diagnostic potential of myocardial early systolic lengthening for patients with suspected non-ST-segment elevation acute coronary syndrome
Source: BMC Cardiovasc Disord. 2023 Jul 19;23:364. doi: 10.1186/s12872-023-03364-y (PMC10357602; doi:10.1186/s12872-023-03364-y)
Supplement: Supplementary file 3 — Additional file 3. Supplemental Table 2. Spearman’s correlations between myocardial deformational parameters. [file 12872_2023_3364_MOESM3_ESM.pdf]

Additional Material

Diagnostic potential of myocardial early systolic lengthening for patients with suspected non-ST-segment elevation acute coronary syndrome

Wanwei Zhang[1], Qizhe Cai[1], Mingming Lin[1], Runyu Tian[1], Shan Jin[1], Yunyun Qin[1,*], Xiuzhang Lu[1,*]

[1]Department of Ultrasound Medicine, Beijing Chao Yang Hospital, Capital Medical University, Beijing, 100020, China.

***Corresponding authors:**

Yunyun Qin, MD, PhD:

Department of Ultrasound Medicine, Beijing Chao Yang Hospital, Capital Medical University, Beijing, 100020, China.

Email: yun_23@126.com

Xiuzhang Lu, MD, PhD:

Department of Ultrasound Medicine, Beijing Chao Yang Hospital, Capital Medical University, Beijing, 100020, China.

Email: echolxz @163.com

Supplemental Table 2. Spearman's correlations between myocardial deformational parameters

Spearman's correlations	**GLS rho**	***P***	**PSI rho**	***P***
DESL	0.17	=0.021	0.39	<0.001
ESI	0.20	=0.005	0.49	<0.001

DESL, duration of early systolic lengthening; ESI, early systolic index; GLS, global longitudinal strain; PSI, post-systolic index.
