# Supplementary material for: Diagnostic potential of myocardial early systolic lengthening for patients with suspected non-ST-segment elevation acute coronary syndrome
Source: BMC Cardiovasc Disord. 2023 Jul 19;23:364. doi: 10.1186/s12872-023-03364-y (PMC10357602; doi:10.1186/s12872-023-03364-y)
Supplement: Supplementary file 4 — Additional file 4. Supplemental Table 3. ROC curve results to predict significant coronary branch stenosis. [file 12872_2023_3364_MOESM4_ESM.pdf]

Additional Material

Diagnostic potential of myocardial early systolic lengthening for patients with suspected non-ST-segment elevation acute coronary syndrome

Wanwei Zhang[1], Qizhe Cai[1], Mingming Lin[1], Runyu Tian[1], Shan Jin[1], Yunyun Qin[1,*], Xiuzhang Lu[1,*]

[1]Department of Ultrasound Medicine, Beijing Chao Yang Hospital, Capital Medical University, Beijing, 100020, China.

***Corresponding authors:**

Yunyun Qin, MD, PhD:
Department of Ultrasound Medicine, Beijing Chao Yang Hospital, Capital Medical University, Beijing, 100020, China.
Email: yun_23@126.com

Xiuzhang Lu, MD, PhD:
Department of Ultrasound Medicine, Beijing Chao Yang Hospital, Capital Medical University, Beijing, 100020, China.
Email: echolxz @163.com

Supplemental Table 3. ROC curve results to predict significant coronary branch stenosis

Variable	Territory perfused by: Nonsignificant stenotic coronary	Territory perfused by: Significant stenotic coronary	AUC	*P*	Cut-off	Sen	Spe
**LAD territories**	*n*=71	*n*=94					
TLS, %	-19.10±3.00	-18.50±3.10	0.55	0.310	-	-	-
PSI, %	1.92 (0.65, 3.87)	2.57 (0.86, 7.44)	0.58	0.076	-	-	-
DESL, ms	6.30 (2.40, 12.60)	8.90 (5.4, 14.80) *	0.60	0.024	6.00	70%	49%
ESI, %	0.40 (0.08, 1.23)	1.08 (0.35, 2.33) *	0.64	0.001	0.34	76%	49%
**LCX territories**	*n*=114	*n*=51					
TLS, %	-16.50±2.60	-15.50±2.90*	0.60	0.040	-15.44	51%	70%
PSI, %	1.49 (0.46, 3.29)	2.22 (0.64, 9.94)	0.59	0.075	-	-	-
DESL, ms	5.00 (0.00, 11.20)	8.60 (5.00, 15.80) *	0.64	0.002	6.80	69%	61%
ESI, %	0.27 (0.00, 1.21)	0.92 (0.12, 2.84) *	0.65	0.001	0.34	75%	53%
**RCA territories**	*n*=111	*n*=54					
TLS, %	-17.90±3.00	-17.40±3.00	0.58	0.102	-	-	-
PSI, %	1.35 (0.42, 2.89)	2.11 (0.74, 5.08) *	0.61	0.024	1.38	69%	51%
DESL, ms	10.60 (4.00, 16.50)	14.20 (8.9, 21.20) *	0.62	0.007	7.30	83%	38%
ESI, %	0.94 (0.15, 2.29)	1.32 (0.58, 3.34) *	0.63	0.004	0.22	93%	30%

ROC, receiver operating characteristic curve; AUC, area under the curve; Sen, sensitivity; Spe, specificity; LAD, left anterior descending artery; TLS, territorial longitudinal strain; PSI, post-systolic index; DESL, duration of early systolic

lengthening; ESI, early systolic index; LCX, left circumflex coronary artery; RCA, right coronary artery. $^{*}P<0.05$ vs. territory perfused by a nonsignificant stenotic coronary.
