# Supplementary material for: Diagnostic potential of myocardial early systolic lengthening for patients with suspected non-ST-segment elevation acute coronary syndrome
Source: BMC Cardiovasc Disord. 2023 Jul 19;23:364. doi: 10.1186/s12872-023-03364-y (PMC10357602; doi:10.1186/s12872-023-03364-y)
Supplement: Supplementary file 5 — Additional file 5. Supplemental Table 4. Interobserver and intraobserver variability (ICCs). [file 12872_2023_3364_MOESM5_ESM.pdf]

Additional Material

Diagnostic potential of myocardial early systolic lengthening for patients with suspected non-ST-segment elevation acute coronary syndrome

Wanwei Zhang[1], Qizhe Cai[1], Mingming Lin[1], Runyu Tian[1], Shan Jin[1], Yunyun Qin[1,*], Xiuzhang Lu[1,*]

[1]Department of Ultrasound Medicine, Beijing Chao Yang Hospital, Capital Medical University, Beijing, 100020, China.

***Corresponding authors:**

Yunyun Qin, MD, PhD:
Department of Ultrasound Medicine, Beijing Chao Yang Hospital, Capital Medical University, Beijing, 100020, China.
Email: yun_23@126.com

Xiuzhang Lu, MD, PhD:
Department of Ultrasound Medicine, Beijing Chao Yang Hospital, Capital Medical University, Beijing, 100020, China.
Email: echolxz @163.com

Supplemental Table 4. Interobserver and intraobserver variability (ICCs)

**Variable**	**Interobserver**	**Intraobserver**
GLS	0.83 (0.68-0.90)	0.95 (0.85-0.99)
PSI	0.62 (0.51-0.69)	0.85 (0.70-0.87)
DESL	0.62 (0.52-0.71)	0.87 (0.76-0.92)
ESI	0.59 (0.48-0.69)	0.85 (0.73-0.91)

ICCs, intraclass correlation coefficients
